# Photoheterotrophy by aerobic anoxygenic bacteria modulates carbon fluxes in a freshwater lake

**DOI:** 10.1038/s41396-021-01142-2

**Published:** 2021-11-20

**Authors:** Kasia Piwosz, Cristian Villena-Alemany, Izabela Mujakić

**Affiliations:** 1 Centre Algatech, Institute of Microbiology, Czech Academy of Sciences, 37981 Třeboň, Czechia; 2 National Marine Fisheries Research Institute, 81-332 Gdynia, Poland; 3 Faculty of Science, University of South Bohemia, 370 05 České Budějovice, Czechia

**Keywords:** Microbial ecology, Limnology, Biogeochemistry

## Abstract

Lakes are a significant component of the global carbon cycle. Respiration exceeds net primary production in most freshwater lakes, making them a source of CO_2_ to the atmosphere. Driven by heterotrophic microorganisms, respiration is assumed to be unaffected by light, thus it is measured in the dark. However, photoheterotrophs, such as aerobic anoxygenic photoheterotrophic (AAP) bacteria that produce ATP via photochemical reactions, substantially reduce respiration in the light. They are an abundant and active component of bacterioplankton, but their photoheterotrophic contribution to microbial community metabolism remains unquantified. We showed that the community respiration rate in a freshwater lake was reduced by 15.2% (95% confidence interval (CI): 6.6–23.8%) in infrared light that is usable by AAP bacteria but not by primary producers. Moreover, significantly higher assimilation rates of glucose (18.1%; 7.8–28.4%), pyruvate (9.5%; 4.2–14.8%), and leucine (5.9%; 0.1–11.6%) were measured in infrared light. At the ecosystem scale, the amount of CO_2_ from respiration unbalanced by net primary production was by 3.69 × 10^9^ g CO_2_ lower over these two sampling seasons when measured in the infrared light. Our results demonstrate that dark measurements of microbial activity significantly bias the carbon fluxes, providing a new paradigm for their quantification in aquatic environments.

## Introduction

Alleviating consequences of the climate change requires a profound understanding of the global carbon cycle. Lakes are an important component of the carbon cycle: their annual burial of organic carbon in sediments exceeds that of oceans [1], but they are still a net source of CO_2_ to the atmosphere at a rate of about 0.9 Pg C y^−1^ (range 0.7–1.3 Pg C y^−1^) [2]. Almost 70% of this surplus CO_2_ production is driven by microbial heterotrophic respiration [3]. Microbial respiration in lakes is predicted to increase as climate change progresses, which may slow down the rate of carbon sequestration [1]. Therefore, an accurate estimate of microbial respiration is crucial for precise global carbon fluxes assessments.

Microbial respiration is typically considered to be independent of light [4]. This assumption has been challenged by the discovery that photoheterotrophic bacteria, such as rhodopsin-containing bacteria and aerobic anoxygenic phototrophic (AAP) bacteria, are abundant in aquatic environments [5, 6]. AAP bacteria use bacteriochlorophyll (BChl)-containing reaction centers to harvest energy from light but cover most of their energy requirements through respiration. Both processes compete for quinone pool on the same membrane. In consequence, respiration gets reduced with the increasing light intensity [7]. Moreover, photochemical ATP production enhances the anabolism, resulting in more efficient incorporation of organic matter and increased bacterial growth efficiency [8, 9].

AAP bacteria comprise up to 30% of total bacteria in the epilimnion during spring and summer [10, 11]. They are on average larger and more active than heterotrophic bacteria [12], exhibit fast growth rates and high susceptibility to grazing, substantially contributing to aquatic food webs and bacterial production [10]. Cultured AAP species exposed to light decrease their respiration by 75% and increase biomass yield by 50% [7, 8]. Light exposure also raises the proportion of active AAP bacteria in freshwater lakes [12]. However, the benefits of light-harvesting by photoheterotrophic bacteria in situ, especially of highly active AAP bacteria, remain unquantified. Measurements of microbial respiration in light are hampered by the oxygen production by photoautotrophic phytoplankton, the inhibitory effect of UV radiation, and the photooxidation of organic matter [13, 14]. Consequently, the rates are typically measured in the dark [15], which biases such estimates in times of an elevated abundance of photoheterotrophs.

This study aimed to quantify the contribution of photoheterotrophic metabolism by AAP bacteria to the carbon fluxes in a freshwater lake. Their BChl-containing reaction centers have an absorption maximum at ~870 nm, which allowed us to use infrared light in the experimental incubations. Phytoplankton and all other microorganisms perceived such conditions as lightless, allowing direct comparison of rates from the IR and conventional dark measurements. We hypothesized that the respiration rate would be lower, while assimilation rates of organic compounds would be higher in the IR light compared to the dark. Moreover, we expected that the effect would vary seasonally with changing environmental conditions and the community composition of all bacteria and of AAP bacteria. We demonstrated that dark measurements of microbial activity significantly bias the estimates of the carbon fluxes in the studied lake, providing a new paradigm for its quantification in aquatic environments.

## Materials and method

### Sampling

Water was sampled from Cep lake in Czechia, at a regular sampling site of 10 m depth (48.944 °N, 14.877 °E). The lake originates from sand mining in the 1970–80s. It is a permanent meso-oligotrophic (chlorophyll-a concentrations ranged from 1.4–16.4 µg L^−1^) seepage reservoir filled with groundwater penetrating from the nearby river Lužnice. The lake area is about 1.16 km^2^, with the maximum depth about 11–12 m. These characteristics are representative for most of temperate and boreal lakes [16].

Samples were collected every four weeks from April till October in 2018, and from April till November in 2019. Ten liters of water were collected from 0.5 m depth using a Ruttner Water Sampler (model 11.003KC Denmark AS). Temperature and oxygen profiles were taken with an EXO1 multi-parameter probe (YSI Inc., Yellow Springs, USA). Water was transported to the laboratory within 2 h from the collection in a closed container made from high-density polyethylene, rinsed three times with the sampled water and stored in a cooled box.

### Nutrients

Samples were filtered through glass fiber filters with 0.4 µm nominal porosity (GF-5, Macherey-Nagel, Düren, Germany). Concentrations of soluble reactive phosphorus (SRP) were determined spectrophotometrically [17, 18]. Concentrations of nitrate and ammonium were measured according to Procházková [19] and Kopáčkek and Procházková [20]. Dissolved organic carbon (DOC) and dissolved nitrogen (DN) were measured with a TOC 5000 A analyzer (Shimadzu, Kyoto, Japan).

### Pigments

Seston from 1.43 to 3.65 L of water was collected onto GF-5 glass fiber filters (diameter 47 mm, Macherey-Nagel). The filters were dried of excess water by gently pressing in a paper towel, and flush frozen in liquid nitrogen. Pigments were extracted and analyzed by HPLC as described in Piwosz et al. [21].

### Net primary production (NPP) and community respiration (oxygen measurements)

Oxygen concentration was measured with the Winkler method [22]. It was chosen because it allows O_2_ concentration to be measured directly in the water without the need to consider carbonic equilibrium, which is the case when changes in CO_2_ concentration is measured [23]. Samples were unfiltered to avoid the removal of particle-associated bacteria and also of free-living AAP bacteria, which tend to be larger than average freshwater bacteria [12]. Glass stoppered Winkler type oxygen bottles (115 mL nominal capacity, VTR glass, Prague, Czechia) were filled with the sampled water directly from the sampler via a rubber tube. Each bottle was first rinsed three times and then filled without the formation of air bubbles. Water was allowed to overflow the neck of the bottle for about 1 min, and the bottle was closed with a glass stopper to avoid air bubbles. The closed bottles were kept in the dark in a cooled box. On the shore, three bottles were selected as T0, and 1.2 mL of manganese (II) chloride solution (concentration 3 mol L^−1^) was addded, followed by the addition of 1.2 mL of a mixture containing 4 mol L^−1^ of sodium iodide solution and 8 mol L^−1^ of sodium hydroxide solution. These samples were processed in the laboratory within 3 h. The remaining bottles were incubated for 24 h at in situ temperature in the IR-box prepared from the MAKROLON IR polycarbonate sheet (4 mm thickness, Professional Plastics, Inc. Fullerton, CA, USA). These panels have a maximum transmittance of 90% in the infrared region 850–2000 nm, 50% at 780 nm, and 0% <740 nm. Dark bottles were wrapped in tinfoil to cut off all irradiance. Illumination was provided with 40 W incandescent (tungsten filament) lightbulbs delivering approx. 50 Wm^−2^ of IR irradiance, which according to our measurements corresponds to IR irradiation on a sunny day. Bottles for NPP were incubated in white light next to the IR-box. To ensure stable temperature and avoid overheating, the incubations were done in a 60 L water bath and the temperature was controlled with a CTB 06 C cryostat (LABIO a.s., Prague, Czechia).

The incubations were terminated by adding manganese (II) chloride and sodium iodide – sodium hydroxide solutions, as described above for the T0 samples. The bottles were incubated for 1 h in a fridge (4 °C). Subsequently, 2.4 ml of 50% H_2_SO_4_ was added, and samples were immediately titrated with 0.01 mol L^−1^ Na_2_S_2_O_3_ solution. The precise concentration of the Na_2_S_2_O_3_ solution was determined each time by titrating the KIO_3_ standard. For the samples collected in 2018, titrations were done manually: the samples were titrated until the solution turned pale yellow. Then, 1 mL of 0.1% starch solution was added, and the titration continued until full decolorization was achieved. In 2019, samples were titrated to a monotonic equivalence point using Metrohm 877 Titrino plus equipped with a double Pt-wire coulometric electrode (Herisau, Switzerland). Respiration rates were calculated as a difference between oxygen concentrations at the end of the incubation and T0 samples.

To calculate the balance between the NPP and respiration measured in the dark and in the IR light, we assumed that one mole of O_2_ produced or consumed was equivalent to one mole of CO_2_. Such assumption may not be always accurate for respiration measurements, but considering the high variability of a respiratory quotient in freshwaters, we decided to adopt the most frequent choice [3]. Values of daily NPP were calculated based on the length of light time from sunrise to sunset on the sampling day (Equation 1 in Supplementary File S1). Values of daily dark respiration were taken directly from the measurements, while values of the daily IR-respiration were calculated as an average weighted for the length of light time to take into account that the effect of light was only during the light time (Equation 2 in Supplementary File S1). Rates were integrated over the duration of the season with water temperatures >10 °C according to our measurements (180 days, Equation 3 in Supplementary File S1). Subsequently, the differences in the Cep Lake’s carbon budget for the surface layer (down to 0.5 m depth) were calculated by multiplying the integrated values by the volume of this layer (Equation 4 and 5 in Supplementary File S1).

### HCO_3_^−^ incorporation

Triplicated water samples (32 mL) were incubated for 3.2–5.2 h in the IR light and the dark at in situ temperature, as described for respiration. Total activity added to each bottle was measured from 1 mL aliquot of the incubated sample that was transferred to a scintillation vial containing 20 μl of 5 mol L^−1^ NaOH (to prevent a loss of ^14^C-bicarbonate). Thirty mL of sample was filtered through 2.5 μm nitrate cellulose filters (Pragopor, Prague, Czechia, diameter 25 mm). Five mL of the filtered water was collected and subsequently filtered through a 0.17 μm nitrate cellulose filter. The resulting cell-free filtrate, which contained ^14^C-DOC was collected. The filtration was done at a low vacuum (0.02 MPa) to avoid cell breakage. The total CO_2_ assimilation rate was calculated as the sum of all these fractions.

The filters were kept in an HCl-saturated atmosphere for 24 h at room temperature in a fume hood. They were placed in scintillation vials and dissolved in 1 mL of ethyl acetate (Penta, Prague, Czechia). Then, 5 mL of Ultima Golt LLT scintillation cocktail (PerkinElmer, Waltham, MA, USA) was added. Five mL of cell-free filtrates were acidified by adding 100 μL 5 mol L^−1^ HCl to volatilize non-incorporated H^14^CO_3_ and incubated 24 h at room temperature in a fume hood. Then, 10 mL of the scintillation cocktail was added. Finally, 5 mL of the scintillation cocktail was added to the total activity samples. Subsequently, the samples were gently mixed and left in the dark for 48 h. The radioactivity in the samples was measured using a Tri-Carb 2810 TR scintillation counter (PerkinElmer).

To estimate carbon fluxes (μmol C L^–1^ h^–1^), a fraction of the added H^14^CO_3_ incorporated or released was corrected for the incubation time and multiplied by the concentration of total dissolved inorganic carbon (DIC). The DIC concentration was calculated based on temperature, pH, and alkalinity measurements (Inolab pH 720, WTW Xylem Inc. Rye Brook, NY, US) determined by Gran titration.

### Assimilation of organic monomers

The difference between microbial activity in the IR light and dark was also estimated based on assimilation rates of radiolabeled glucose, pyruvate, leucine and thymidine (American Radiolabeled Chemicals, St. Louis, MO, USA). Tritiated glucose (specific activity (SA): 2220 GBq mmol^−1^), leucine (SA: 4440 GBq mmol^−1^) and thymidine (SA: 2275.5 GBq mmol^−1^) were added to 5 mL samples to a final concentration of 5 nmol L^−1^. ^14^C-pyruvate (SA: 2.035 GBq mmol^−1^) was added to a final concentration of 10 nmol L^−1^. Trichloroacetic acid (TCA) was added to the killed controls to a final concentration of 1%. Samples were incubated for 1 h in the dark and IR light as described for respiration. The incubations were terminated as the killed controls and kept at 4 °C in the dark until processed within <4 h. Biomass was collected onto 0.17 μm nitrate cellulose filters as described for HCO_3_^−^ incorporation. The filters were washed twice with 2.5 mL of ice-cold 5% TCA, and then twice with 2.5 mL of ice-cold 80% ethanol [24]. They were placed in the scintillation vials and air-dried overnight. Dried filters were dissolved in 1 mL of ethyl acetate, and 5 mL of Ultima Golt LLT scintillation cocktail (PerkinElmer) was added. Samples were gently mixed and left in the dark for 48 h. The radioactivity in the samples was measured using a Tri-Carb 2810 TR scintillation counter (PerkinElmer).

### Bacterial and AAP abundance

Samples of 50 mL were fixed with buffered, sterile-filtered paraformaldehyde (Penta, Prague, Czechia) to a final concentration of 1%, and 0.5 mL was filtered onto white polycarbonate filters (pore size 0.2 µm, Nucleopore, Whatman, Maidstone, UK). Cells were stained with 4’,6-diamidino-2-phenylindole (DAPI) at concentration of 1 mg L^−1^ [25]. Total and AAP bacterial abundances were determined using an epifluorescence Zeiss Axio Imager.D2 microscope equipped with Collibri LED module illumination system (Carl Zeiss, Jena, Germany). Ten microphotographs were taken for every sample under 325–370 nm excitation and 420–470 nm emission wavelengths for DAPI fluorescence (total bacteria), 450–490 nm excitation and 600–660 nm emission wavelengths for autofluorescence from Chl-*a* (algae and cyanobacteria), and combined 325–370 nm, 450–490 nm, 545–565 nm and 615–635 nm excitation and 645–850 emission wavelengths for autofluorescence from BChl-*a* (AAP bacteria). As some part of Chl-*a* autofluorescence is also visible in the infrared spectrum, only the IR-positive cells that did not show any autofluorescence from Chl-*a* were counted as AAP bacteria [26].

### DNA extraction

Between 300 and 1460 mL of water were filtered through sterile 2 µm and 0.2 µm Nucleopore Track-Etch Membrane filter units (Whatman). The filters were put inside sterile cryogenic vials (Biologix Group Limited, Jinan, Shandong China) containing 0.55 g of sterile zirconium beads, flash-frozen in liquid nitrogen and stored at −80 °C. Total nucleic acids were extracted within a month following the protocol by Nercessian et al. [27]. Lysis buffer (75 µl of 10% sodium dodecyl sulfate (Tokyo Chemical Industry CO, LTD., Tokyo, Japan), 75 µl of 10% N-Lauroylsarcosine (Sigma–Aldrich, St. Louis, USA), 750 µl of phenol-chloroform-isoamyl alcohol (25:24:1; AppliChem GmbH, Darmstadt, Germany) and 750 µl of 10% hexadecyltrimethylammonium bromide (CTAB; Sigma-Aldrich) in 1.6 M NaCl and 240 mM potassium-phosphate-buffer, pH = 8) was added to the vials and they were vortexed for 10 min. After centrifugation for 10 min at 4 °C and 16,000  x g, supernatant was mixed carefully with the equal volume of chloroform (PENTA s.r.o., Prague, Czechia). After the second centrifugation, the supernatant was mixed with two volumes of 30% Poly(ethylene glycol) (PEG; Sigma–Aldrich) in 1.6 M of NaCl, and incubated for 2 h in the dark at 4 °C, followed by centrifugation for 90 min at 4 °C and 17,000  x g. The pellet was washed with 70% ethanol (VWR International S.A.S., Fontenay-sous-Bois, France) and centrifuged again for 1 min. Extracted DNA was re-suspended in 35 µl of DNase and RNase-free water (MP Biomedicals, Solon, OH, USA) and stored at −20 °C. Concentration and quality of the extracts were checked using NanoDrop (Thermo Fisher Scientific).

### Bacterial community composition

The V3-V4 region of bacterial 16S rRNA gene was amplified using 341F and 785R primers [28]. PCR was performed in triplicate 20 μL reactions using Phusion™ High-Fidelity DNA Polymerase (Thermo Scientific, USA) with the following reaction conditions: 98 °C for 3 min, 25 cycles at 98 °C for 10 s, 60 °C for 20 s, 72 °C for 20 s, and a final extension at 72 °C for 3 min. The triplicate product reactions for each sample were pooled andpurified from the gel using the kit Wizard SV Gel and PCR Clean-Up System (Promega, USA), and sequenced on Illumina MiSeq (2 × 250 bp) platform of the Genomic Service of the Universitat Pompeu Fabra (Barcelona, Spain).

Initial analysis, performed as described below, indicated that the communities in both fractions were similar for each sampling day (Bray-Curits similarity >65% except for the samples from 9th May and 29th Aug 2018 (10% each), 1st Aug 2018 (21%), 25th Nov 2018 (1%), and 14th Aug 2019 (22%), Supplementary Fig. S1A). Thus, we decided to concatenate the fastaq files and analyze both fractions together as the total community. This also facilitated statistical analysis, as the activity rates were measured for the whole community without fractionations.

Reads quality was evaluated using FastQC v0.11.7 (Babraham Bioinformatics, Cambridge, UK). After primer sequences trimming using Cutadapt [29] (v1.16), the number of reads per sample ranged from 49,354 to 188,942. Subsequent analyses were done in the R/Bioconductor environment using the dada2 package (version 1.14.1) [30]. Forward and reverse reads were truncated to 225 bp and low quality sequences were filtered out with the filterAndTrim function (truncLen = c(225, 225), maxN = 0, maxEE = c(2, 2), truncQ = 2), which reduced the number of reads per sample to range from 30,190 to 143,552. After merging and chimera removal using the removeBimeraDenovo function, 4,893 amplicon sequence variants (ASV) were obtained. Rare ASVs (not seen >3 times in at least 20% of the samples) were removed, which reduced the number of ASVs to 658, and the number of reads to 14,613–69,046 per sample. Taxonomic assignment was done using SILVA 138.1 database [31, 32] released on August 27, 2020. ASVs identified as Chloroplast or Cyanobacteria were excluded from the analyses, giving the final number of 546 ASVs and from 10,819 to 54,799 reads per sample. The bacterial community composition graphs were done using phyloseq [33] and ggplot2 [34] packages.

### AAP community composition

The composition of AAP community was analyzed by amplicon sequencing of *pufM* gene encoding the M subunit of bacterial type-2 reaction centers. This gene is routinely used for diversity studies of AAP bacteria [35].

*Puf*M gene amplicons (approx. 245 bp) were prepared using pufM_UniF (5′-GGN AAY YTN TWY TAY AAY CCN TTY CA-3′) and pufM_WAW (5′-AYN GCR AAC CAC CAN GCC CA-3′) primers [36]. PCR was performed in triplicate 20 μL reactions using Phusion™ High-Fidelity DNA Polymerase (Thermo Scientific, USA) with the following reaction conditions: 98 °C for 3 min, 27 cycles at 98 °C for 10 s, 58 °C for 30 s, 72 °C for 30 s, and a final extension at 72 °C for 5 min. The triplicate product reactions for each sample were pooled and gel purified using the kit Wizard SV Gel and PCR Clean-Up System (Promega, USA). The sequencing was performed on the Illumina MiSeq platform (2 × 150 bp) at Macrogen, South Korea.

The fastq files were concatenated as described for bacteria communities. The Bray-Curtis similarity between two fractions for each sampling day was >70%, except for the samples from 1st Aug 2018 (47%) and 14th Aug 2019 (18%, Supplementary Fig. S1B).

The samples were analyzed as described for bacterial communities. The number of reads per sample ranged from 192,360 to 239,418 after the cutadapt trimming. Forward and reverse reads were truncated to 130 bp, and the number of reads per sample after the quality filtering and denoising ranged from 189,432 to 235,311. Merging the forward and reverse reads with mergePairs function created 12,692 ASVs and reduced the number of reads to 183,136–221,281 per sample. The chimera removal lowered the number of ASVs to 1816, and the number of reads to 159,451–208,679. Rare ASVs (not seen >3 times in at least 20% of the samples) were removed, which resulted in the final 566 ASVs, and a number of reads ranging from 155,915 to 203,021 per sample. A manually curated taxonomic database was used for taxonomic assignment following the naïve Bayesian classifier method [37]. It contained 1580 unique *puf*M sequences, downloaded from the Fungene repository on May 16, 2019 (http://fungene.cme.msu.edu [38]), from metagenomes from the Římov Reservoir [39, 40] and from the Genome Taxonomy database accessed on September 16, 2020 [41].

### Statistical analysis

Linear mixed-effects models were calculated in R (version 3.6.2) using lme function from the nlme package (version 3.1.143) on untransformed activity data and log_10_ transformed environmental variables [42]. Models’ parameters were estimated using maximum likelihood method and their significance was tested with ANOVA. Relationships between the activity measures, the environmental variables and the composition of AAP communities were investigated with distance-based linear models (DistLM) [43, 44] in Primer (version 7.0.13) with PERMANOVA + 1 add on (e-Primer, Plymouth, UK) [45]. The sequence reads were transformed with the varianceStabilizingTransformation function of the DESeq2 package [46] (version 1.14.1, blind = FALSE, fitType = “mean”).

### Data accessibility

The sequences of 16S and pufM amplicons that support the findings of this study have been deposited in the EMBL database as the BioProject with the accession number PRJEB41596, together with most of the environmental metadata. The scripts and the remaining data supporting the results are included in the Supplementary Material.

## Results and discussion

**Fig. 1:**
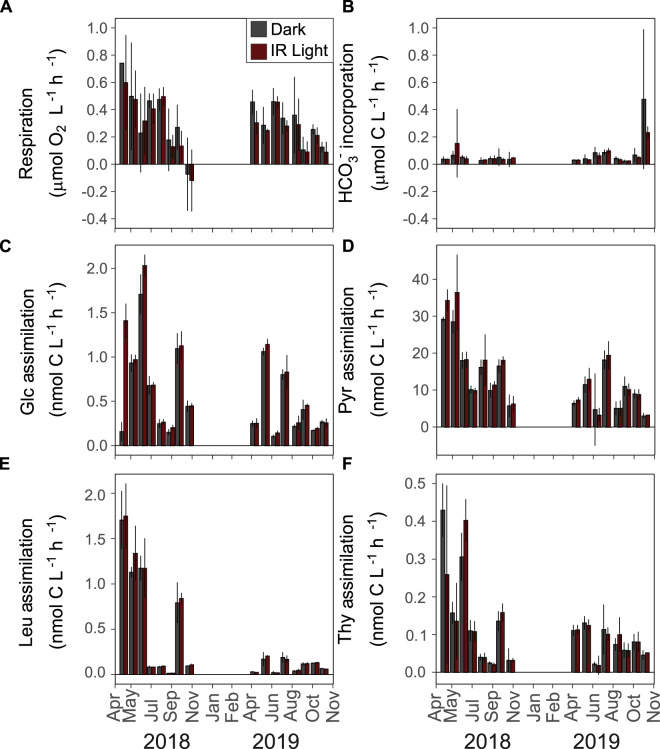
Microbial activity in the surface layer of Lake Cep measured in the dark (dark gray) and the IR light (dark red). **A** respiration rate; (**B**) incorporation rate of H^14^CO_3_^−^; (**C**) assimilation rate of glucose; (**D**) assimilation rate of pyruvate; (**E**) assimilation rate of leucine; (**F**) assimilation rate of thymidine. Bar plots show mean values of triplicate measurements; error bars indicate 95% confidence intervals. Scales on *Y* axes differ between the panels except for panels (**A**) and (**B**).

Microbial activity was the highest during spring phytoplankton maxima, and in summer at temperatures >20 °C (Fig. 1 and Supplementary Fig. S2). The difference between the activity rates measured in the dark and IR light varied over the sampling period and were the highest in April and May in both years (Fig. 1).

**Fig. 2:**
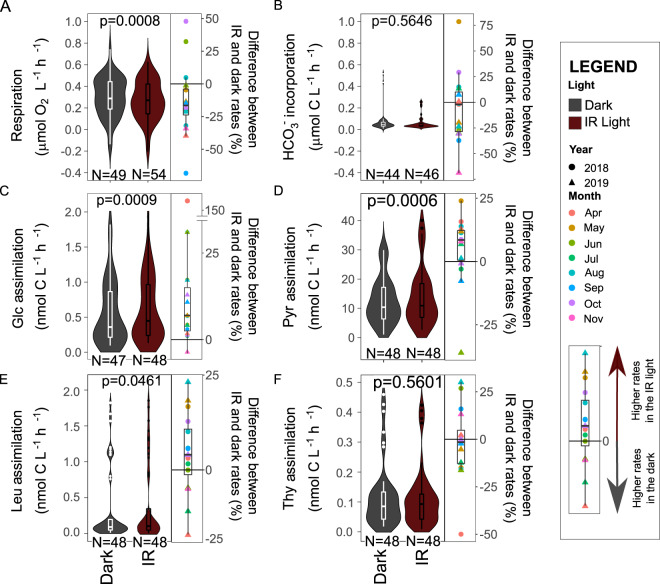
Difference between microbial activity measured in the dark and IR light. Violin plots with a box-plot inside compare the distribution of the data for the dark and IR measurements (p: *p* value from the ANOVA test of significance of the linear mixed-effects model with the date as the random variable. N: number of independent measurements). Box-plots show percent difference between mean rates measured in the dark and in the light (*N* = 16) for each sampling day. **A** respiration rate; (**B**) incorporation rate of H^14^CO_3_^−^; (**C**) assimilation rate of glucose; (**D**) assimilation rate of pyruvate; (**E**) assimilation rate of leucine; (**F**) assimilation rate of thymidine.

The average respiration rate was 15.2% (95% CI: 6.6–23.8%, *p* value = 0.0008) higher in the dark (average (95% CI): 0.30 (0.25–0.35) μmol O_2_ L^−1^ h^−1^) than in the IR light (0.27 (0.22–3.20) μmol O_2_ L^−1^ h^−1^; Fig. 2A). To exclude the possibility that the observed difference was due to oxygen production by phytoplankton, we also measured incorporation of H^14^CO_3_^−^ in the dark and the IR light. The bicarbonate incorporation rates were about 5-fold lower than respiration rates (Fig. 1B) and did not differ significantly between the dark and IR light (*p* value = 0.5646, Fig. 2B). Thus, the lower respiration rates measured in the IR light can be attributed to the photoheterotrophic activity of AAP bacteria and not due to oxygen production by phytoplankton.

The decrease in the respiration rate in the IR light compared to dark was driven by production of ATP from the light energy harvested by the reaction centers, which results in a more efficient metabolism as the organic carbon can be redirected from catabolism to anabolism [7]. Consequently, increased assimilation rates of sources of organic carbon should be observed in the IR light. Such differences ought to be especially conspicuous for molecules that can be directly channeled either to anabolic or catabolic reactions depending on the cells’ requirements for ATP production, e.g., glucose and pyruvate [8]. In agreement with this prediction, the average assimilation rate of glucose was 18.1% (7.8–28.4%, *p* value = 0.0009) higher in the IR light (0.67 (0.51–0.82) nmol C L^−1^ h^−1^) than in the dark (0.55 (0.42–0.68) nmol C L^−1^ h^−1^; Fig. 2C). The most noticeable difference was measured in April 2018, when the assimilation rate in the IR light was 9-fold higher than in the dark (Fig. 1C). Likewise, the assimilation rate of pyruvate was enhanced by 9.5% (4.2–14.8%; *p* value = 0.0006) in the IR light, and averaged at 13.94 (11.10–16.78) nmol C L^−1^ h^−1^ for the IR light, and at 12.67 (10.40–14.95) nmol C L^−1^ h^−1^ for the dark measurements (Fig. 2D).

We also compared assimilation rates of leucine (Fig. 1E). This molecule is used by bacteria for protein synthesis and is routinely applied to measure bacterial secondary production in aquatic environments [24]. The average assimilation rate of leucine in the IR light (0.39 (0.23–0.55) nmol C L^−1^ h^−1^) was 5.9% (0.1–11.6%; *p* value = 0.0461) higher than in the dark (0.36 (0.20–0.51) nmol C L^−1^ h^−1^, Fig. 2E). This further supports the notion that the lower respiration rates in the IR light allowed for more efficient metabolism and increased biomass production by the microbial community. Finally, we also used thymidine (Fig. 1F), which is routinely applied to measure rates of DNA synthesis and to determine growth rates of aquatic bacteria [47]. The exposure to the IR light did not significantly affect thymidine assimilation rates (*p* value = 0.5601), which were even slightly lower in the IR (0.11 (0.08–0.14) nmol C L^−1^ h^−1^) than in the dark conditions (0.12 (0.09–0.15) nmol C L^−1^ h^−1^, Fig. 2F). This agrees with the previous observations that the thymidine assimilation rate is independent of irradiance in a cultured AAP species [8], and might result from the fact that DNA synthesis occurs in evening hours [48].

**Fig. 3:**
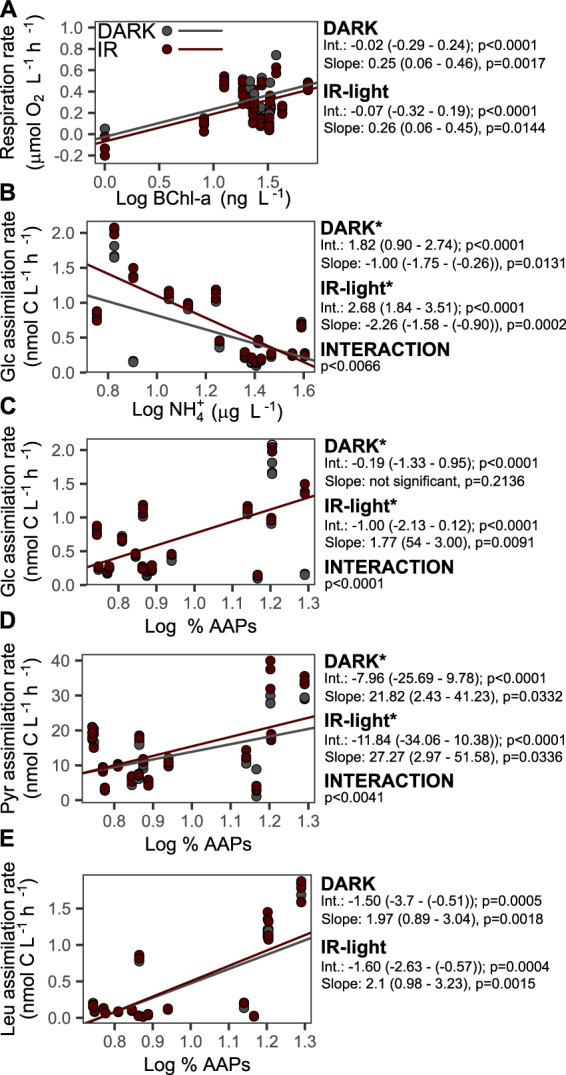
Correlations between microbial activity rates and environmental variables. Average and 95% confidence intervals are given for intercept (Int.) and slope values. Star indicates an interaction with the light (i.e. significantly different correlation coefficients for the rates measured in the dark and the IR light). Correlations between (**A**) concentrations of bacteriochlorophyll-a and respiration rate; (**B**) concentrations of ammonia and assimilation rates of glucose; and between relative abundance of AAP bacteria and assimilation rate of glucose (**C**), pyruvate (**D**) and leucine (**E**).

The effect of the IR light on microbial activity, as compared to dark measurements, varied seasonally (Fig. 1). Such dynamics in aquatic environments often correlate with environmental variables, such as temperature [11]. Thus, we augmented the linear mixed-effects models with measured physico-chemical (temperature, pH, inorganic N and P, dissolved organic carbon), and biological (bacterial and AAP numbers, photosynthetic pigments) variables (Supplementary Fig. S2) to test their correlations with the activity rates, and also to test whether such relationship had been affected by light. Respiration rates, both those measured in the dark and in the IR light, correlated positively only with BChl-*a* concentrations (Fig. 3A), indicating overall high contribution of BChl-*a* containing AAP bacteria to the total community respiration, a phenomenon observed also in other lakes [11, 12]. The slope of this relationships was similar for the dark and the IR rates, likely reflecting the fact that the rate of ATP production by photophosphorylation depends, among others, on the number of BChl-*a* containing reaction centers that generate electrons flow [8]. AAP species differ with regard to the number of BChl-*a* molecules in reaction centers [49], which may explain the lack of significant correlation between the concentrations of BChl-*a* and the absolute abundances of AAP bacteria (Pearson correlation test, *p* value = 0.05902, R^2^ = 0.4665878, *t* = 2.0431), and the concentrations of BChl-*a* and the relative abundances of AAP bacteria (*p* value = 0.07377, R^2^ = 0.4445804, *t* = 1.9223). Therefore, the lack of direct relationship between the respiration rates and AAP bacteria abundance does not contradict our conclusion on their importance in total microbial respiration.

Assimilation rate of glucose negatively correlated with concentrations of ammonia, however, the correlation was stronger for the rates measured in IR light than those measured in the dark (Fig. 3B). This indicates that although such relationship was important for both heterotrophic and photoheterotrophic bacteria assimilating glucose, it affected AAP bacteria more strongly. In agreement with this interpretation, glucose assimilation rates measured in the IR light (but not in the dark) correlated positively with the relative abundance of AAP bacteria (Fig. 3C). Similarly, the correlations between the relative AAP abundance and assimilation rates of pyruvate (Fig. 3D) differed for dark and IR light measurements. The significantly steeper slopes of these correlations for the rates measured in IR light indicate not only the overall high importance of AAP bacteria for assimilation of glucose and pyruvate, but also that their contribution was additionally enhanced in the light. This supports attributing the observed differences between the dark and the IR rates of microbial activities to photoheterotrophic metabolism by AAP bacteria.

**Fig. 4:**
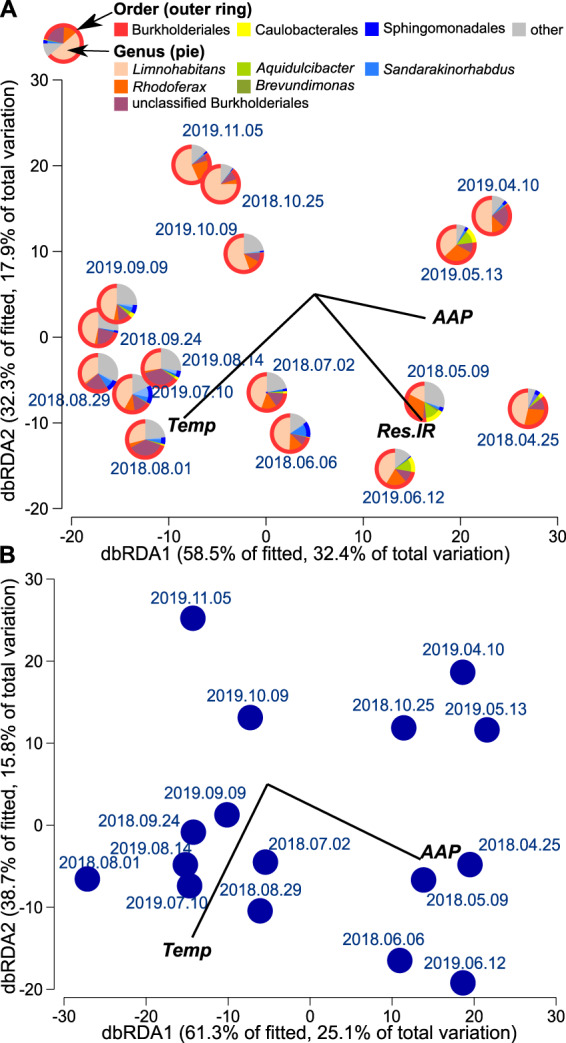
Relationships between bacterial and AAP community composition and environmental variables. Distance-based redundancy analysis biplots relating the observed variability in the composition of communities of AAP (**A**) and all bacteria (**B**) to the explanatory variables (black lines). The pie charts in the panel (**A**) show relative abundance of the top three AAP orders (bar plots showing composition of AAP and bacterial communities can be found in Supplementary Figs. S3 and S4). AAP: abundance of AAP bacteria, Res.IR: respiration rate in the IR light, Temp: water temperature.

Interestingly, while catabolic processes correlated with BChl-*a* concentration, anabolic were related with relative abundance of AAP bacteria. The former relationship can result from direct dependence of photophosphorylation rate on electrons production on reaction centers that supplements the catabolic ATP production via respiration [7, 8], as explained above. On the other hand, the positive relationship between the anabolic metabolism and the relative abundance of AAP bacteria might indicate their generally high ability to assimilate organic compounds. The use of organic molecules substantially varies between different bacterial lineages [50]. Even closely related strains of an important freshwater AAP species *Limnohabitans planktonicus*, II-D5 and 2KL-16 [51], show different growth response to the same organic substrates [52]. This indicates that in situ microbial photoheterotrophic activity may also depend on AAP community composition [21, 53], which was tested statistically. The distance based linear model (DistLM) that best explained variability in the AAP community composition included AAP abundance (*p* = 0.0025, Pseudo-F = 3.9203, 21.9% of the explained variability), water temperature (*p* = 0.0001, Pseudo-F = 4.8571, 21.2% of the explained variability), and respiration rate measured in the IR light (*p* = 0.0005, Pseudo-F = 3.3375, 12.4% of the explained variability). The respiration rate measured in the dark was not statistically significant factor in any of the calculated models, even when the rates in the IR light were intentionally excluded from the analysis. Higher AAP abundance and respiration rate in the IR light were associated with an increased contribution of alphaproteobacterial orders Caulobacterales (genus *Aquidulcibacter*) and Sphingomonadales (genus *Sandarakinorhabdus*) and genus *Rhodoferax* (Burkholderiales, Gammaproteobacteria) to AAP communities (Fig. 4A). In contrast, genus *Limnohabitans* and other unclassified Burkholderiales [54] showed higher relative abundance  in summer or in autumn, when the differences between the microbial activity measured in the dark and in the IR were the lowest (Fig. 1, Fig. 4A, Supplementary Fig. S3). The observed variability in the bacterial community was explained in 23.1% by the abundance of AAP bacteria (*p* = 0.0006, Pseudo-F = 4.2097) and in 17.8% by temperature (*p* = 0.0001, Pseudo-F = 3.9078; Fig. 4B). This confirms the overall importance of photoheterotrophy in the community metabolism, extend of which depends not only on the abundance of AAP bacteria, but also on the taxonomic composition of their communities.

The key result of our study is that the dark incubations substantially overestimate microbial respiration and underestimate microbial production rates in lake surface waters, challenging a current paradigm for measuring microbial activity in aquatic environments. Moreover, we demonstrated high importance of photoheterotrophy by AAP bacteria in freshwater pelagic environments, confirmed over two years using different, independent measures of microbial in situ activity, and pointed to the factors that may drive it. However, AAP bacteria are not the only photoheterotrophic microorganisms in freshwater lakes. Many bacteria produce ATP in light using rhodopsin. Actinobacteriota from acI clade, a group that often dominates bacterial communities in freshwater lakes [55], contains actinorhodopsin [56, 57] that have ion-pumping activity in green light and are highly expressed in situ [58]. The net energy gain from rhodopsin-based phototrophy is lower than from bacteriochlorophyll-based photosystems of the AAP bacteria [59]. Nevertheless, their contribution to the lake photoheterotrophy might have been substantial, as Actinobacteriota were a dominant group of bacteria in both sampling season (Supplementary Fig. S4A). Unfortunately, actinorhodopsins absorb green light that is also used by oxygenic photoautotrophs. Thus, their photoheterotrophic activity cannot be as easily measured by applying a specific light wavelength as in the case of AAP bacteria. In our previous experiment, we used (3-(3,4-dichlorophenyl)−1,1-dimethylurea) to block photosystem II in oxygenic phototrophs without affecting bacteria [21]. However, a strong inhibition of the dark bacterial respiration was observed in this treatment, indicating some detrimental effect on bacterial activity and hampering the use of such approach to environmental studies. Picocyanobacteria are another group of microorganisms that assimilate amino acids in the dark, as observed for marine *Prochlorococcus* [60]. This does not seem to be the case for picocyanobacteria in lakes [50], whose communities in our study were dominated by an obligate photoautotroph *Cyanobium* [61]. Finally, the fact that we had to use IR light to avoid confounding effect from oxygenic photosynthesis might have lowered photoheterotrophy even by AAP bacteria, as they can also use light in the visible spectrum (400–600 nm). Taken together, our results are the lower estimates for the importance of the photoheterotrophy in freshwater lakes.

**Fig. 5:**
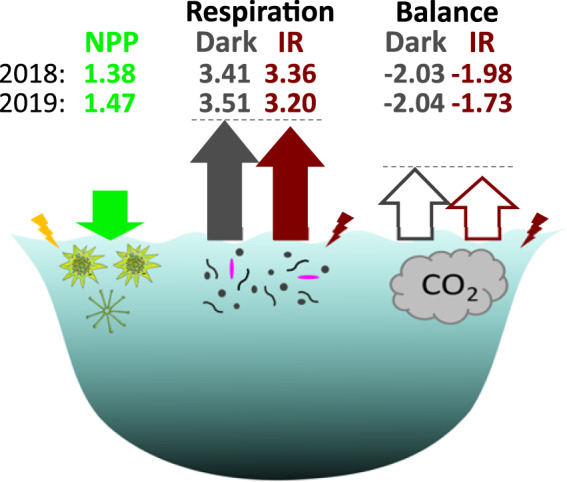
Comparison of carbon fluxes for the surface layer of the Lake Cep measured in the dark and the infrared light. CO_2_ fluxes (10^10^ g CO_2_ over the season with water temperature >10 °C) from net primary production (NPP, green color) and respiration (Res) for the surface layer (0.5 m) of the whole area of the lake Cep based on dark (gray color) and infrared (red color) measurements. Lengths of the arrows are scaled to reflects the differences, and the dash lines are meant to aid direct comparison between the arrows. The difference between the excessive CO_2_ from the respiration based on dark vs infrared (IR) measurements was 0.54 × 10^9^ g CO_2_ in 2018 and 3.15 × 10^9^ g CO_2_ in 2019, summing up to 3.69 × 10^9^ g CO_2_ over these two years. Lightning indicate energy from light, pink microbe indicates AAP bacteria.

Nevertheless, they alter the current understanding of carbon fluxes at the ecosystem level. For instance, the balance between the net primary production (NPP) and bacterial respiration (BR) determines whether a system is heterotrophic, i.e., releases CO_2_ to the atmosphere (NPP < BR), or autotrophic, i.e., absorbs CO_2_ from the atmosphere (NPP > BR). This balance is negative for most freshwater lakes, making them an important source of CO_2_ to the atmosphere at the global scale [1]. However, as we have shown here, dark measurements overestimate microbial respiration, resulting in biased carbon budget for lakes. Calculations of the carbon fluxes for the surface layer of the Lake Cep indicated that it is heterotrophic both in case of dark and IR measurements (Fig. 5). However, the amount of excessive CO_2_ during months with water temperature >10 °C (April-October) was significantly lower for the respiration measurements in the IR light compared to dark (*p* value = 0.0255). The estimates of CO_2_ released during these months based on the IR measurements were lower by 0.54 × 10^9^ g CO_2_ in 2018 and by 3.15 × 10^9^ g CO_2_ in 2019 (Fig. 5). These numbers illustrate the potential impact of photoheterotrophic activity by AAP bacteria in freshwater lakes. More measurements of microbial activity in the IR light instead of in the dark, and especially in habitats where AAP bacteria are abundant, such as the coastal areas of the ocean or mountain lakes [35], will improve estimates of microbial respiration and production, providing a comprehensive understanding of the role of photoheterotrophy in the global carbon cycle.

## Supplementary information

Supplemental Material

Dataset S1

Dataset S2

Dataset S3

Dataset S4

Dataset S5

Dataset S6

Dataset S7
