# Supplementary material for: Photoheterotrophy by aerobic anoxygenic bacteria modulates carbon fluxes in a freshwater lake
Source: ISME J. 2021 Nov 20;16(4):1046–54. doi: 10.1038/s41396-021-01142-2 (PMC8941148; doi:10.1038/s41396-021-01142-2)
Supplement: Supplementary file 1 — Supplemental Material [file 41396_2021_1142_MOESM1_ESM.pdf]

Supplementary Materials for

Photoheterotrophy by aerobic anoxygenic bacteria modulates carbon fluxes in a freshwater lake

Kasia Piwosz[1,2*], Cristian Villena-Alemany[1,3], Izabela Mujakić[1,3]

[1]Centre Algatech, Institute of Microbiology, Czech Academy of Sciences, 37981 Třeboň, Czechia

[2]National Marine Fisheries Research Institute, 81-332 Gdynia, Poland.

[3]Faculty of Science, University of South Bohemia, 370 05 České Budějovice, Czechia

* corresponding author: Kasia Piwosz, e-mail: kpiwosz@mir.gdynia.pl

This file includes:

Supplementary File S1. Calculations of the carbon budgets for Figure 5. 4

Equation 1. Scaling measured net primary production rate to the day light time 4

Equation 2. Scaling measured respiration rate in the IR light to the day light time 4

Equation 3. Integrating NPP_{DL}, Res_M-Dark and Res_{DL}-IR over the season with temperatures > 10°C (April-October, 180 days). 4

Equation 4. Upscaling NPP_{INT}, Res_{INT}-Dark and Res_{INT}-IR to the volume of the surface layer of the whole lake. 5

Equation 5. Recalculating µmol O_2 to g CO_2 assuming respiration quotient 1 5

Supplementary Figure S1. Nonmetric multidimensional scaling plots of total (A) and AAP (B) bacterial communities in different size fractions. 6

Supplementary Figure S2. Environmental variables. 7

Supplementary Figure S3. Composition of AAP bacteria communities (based on the *pufM* gene amplicons). 8

Supplementary Figure S4. Composition of bacterial communities (based on the 16S rRNA gene amplicons). 9

R scripts for data analysis 10

Script for calculating linear mixed-effects model 10

Script for 16S amplicon analysis 11

Script for AAP bacteria analysis using PufM gene sequences 12

Script for transformation of read number data for subsequent distance based redundancy analysis in Primer7 with PERMANOVA+ addon 13

Legends for Datasets S1 to S7 13

Supplementary File S1. Calculations of the carbon budgets for Figure 5.

Calculation were based on mean values of triplicate measurements. Net primary production (NPP) was calculated from oxygen production over 24 h in the white light, respiration in the dark (Res-Dark) and in the infrared light (Res-IR) from oxygen consumption over 24 h in the dark and IR light, respectively (detail description of the method is in the section Material and methods: Net primary production (NPP) and community respiration (oxygen measurements))). Respiration quotient of 1 was assumed.

Equation 1. Scaling measured net primary production rate to the day light time

$$NPP_{DL} = \frac{\mathrm{DL}}{24} \times NPP_{M}$$

where:

NPP_{DL}: NPP during the day light time, in µmol O_2 L^{-1} d^{-1}

DL: day light time (hours) from the sunrise to the sunset on the day when the measurements were conducted

NPP_M: net primary production measured over 24 h

Equation 2. Scaling measured respiration rate in the IR light to the day light time

$$Res_{DL}\text{-}IR = \frac{\mathrm{DL}}{24} \times \mathrm{Res}_{M}\text{-IR} + \left(1 - \frac{\mathrm{DL}}{24}\right) \times \mathrm{Res}_{M}\text{-Dark}$$

where:

Res_{DL}-IR: Res-IR during the day light time, in µmol O_2 L^{-1} d^{-1}

DL: day light time from the sunrise to the sunset on the day when the measurements were conducted

Res_M-IR: Respiration measured in the IR light measured over 24 h

Res_M-Dark: Respiration measured in the IR light measured over 24 h

Equation 3. Integrating NPP_{DL}, Res_M-Dark and Res_{DL}-IR over the season with temperatures > 10°C (April-October, 180 days).

$$NPP_{INT} = \sum \frac{NPP_{DL}(T_n) + NPP_{DL}(T_{n+1})}{T_{n+1} - T_n}$$

where:

NPP_{INT}: NPP integrated over the sampling period, in µmol O_2 L^{-1} 180 d^{-1}

NPP_{DL}: NPP during the day light time, in µmol O_2 L^{-1} d^{-1}

T_n: day of the year on which NPP_{DL} was measured

T_{n+1}: day of the year on which NPP_{DL} was measured, directly following T_n

Integrated respiration in the dark (Res_{INT}-Dark) and in the IR light (Res_{INT}-IR) was calculated analogically. For Res_{INT}-Dark, Res_{DL}-Dark was equal to Res_M-Dark. The calculation were done separately for each year

Equation 4. Upscaling NPP_{INT}, Res_{INT}-Dark and Res_{INT}-IR to the volume of the surface layer of the whole lake.

$$NPP = NPP_{INT} \times V_{0.5\,m}$$

where:

NPP: NPP in the surface layer (upper 0.5 m) of the studied lake integrated over the sampling period, in µmol O_2 180 d^{-1}

NPP_{INT}: NPP integrated over the sampling period, in µmol O_2 L^{-1} 180 d^{-1}

$V_{0.5m}$: Volume of the surface layer (upper 0.5 m) of the Lake Cep, calculated from the lake area of 1 162 407 m^2.

Res-Dark and Res-IR were calculated analogically.

Equation 5. Recalculating µmol O_2 to g CO_2 assuming respiration quotient 1

$$\text{g CO}_2 = \mu\text{mol O}_2 \times 0.044$$

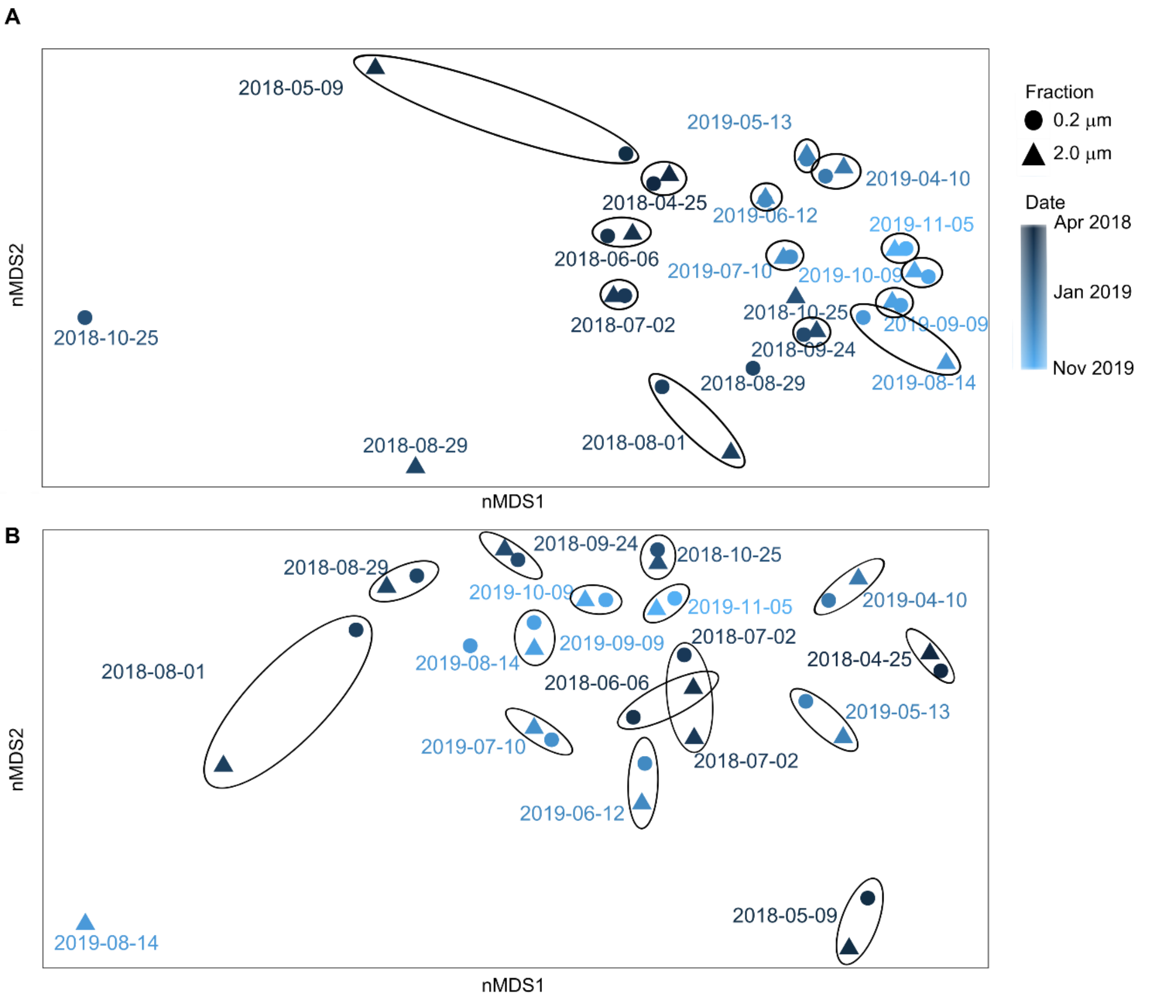

Supplementary Figure S1. Nonmetric multidimensional scaling plots of total (A) and AAP (B) bacterial communities in different size fractions.

Plots were calculated based on Bray-Curtis distances.

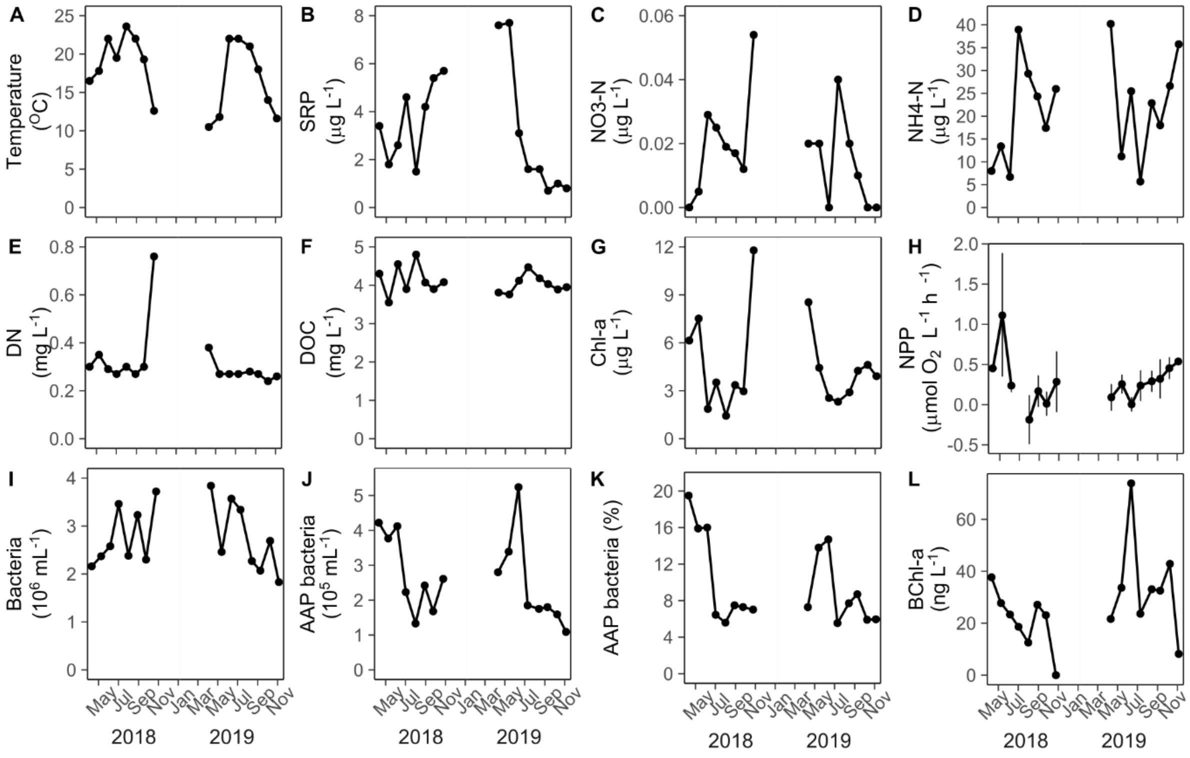

Supplementary Figure S2. Environmental variables.

A: temperature; **B**: concentration of soluble reactive phosphorus (SRP); **C**: concentration of nitrogen in form of nitrate (NO3-N); **D:** concentration of nitrogen in form of ammonia (NH4-N); **E:** concentration of dissolved nitrogen (DN); **F:** concentration of dissolved organic carbon (DOC); **G:** concentration of chlorophyll-a (Chl-a); **H:** Net primary production (NPP). Dots show mean values of triplicate measurements, error bars indicate 95% confidence intervals; **I:**, abundance of all bacteria; **J:** abundance of APP bacteria; **K:** relative abundance of AAP bacteria; **L**: concentration of bacteriochlorophyll-a (BChl-a)**.**

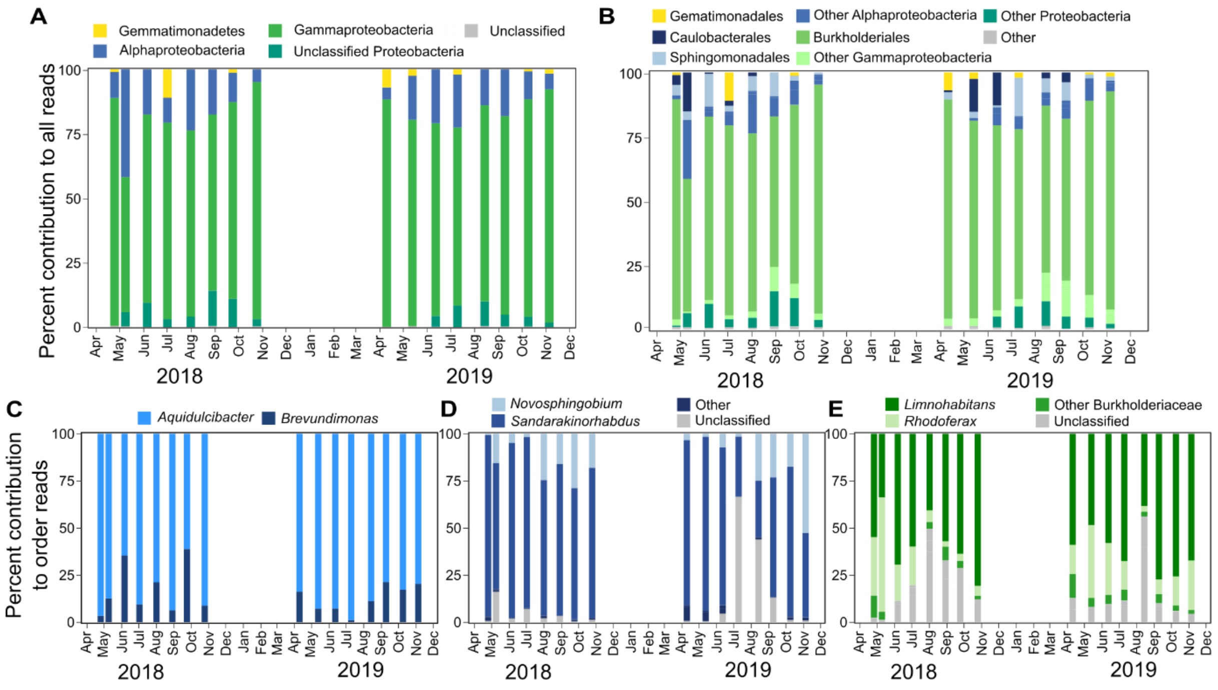

Supplementary Figure S3. Composition of AAP bacteria communities (based on the *pufM* gene amplicons).

A: percent contribution of classes to the total number of reads in the sequencing libraries; **B:** percent contribution of orders to the total number of reads in the sequencing libraries; **C:** percent contribution of genera to the number of reads coming from order Caulobacteriales (Alphaproteobacteria); **D:** percent contribution of genera to the number of reads coming from order Shpingomonadales (Alphaproteobacteria); **E:** percent contribution of genera to the number of reads coming from order Burkholderiales (Gammaproteobacteria).

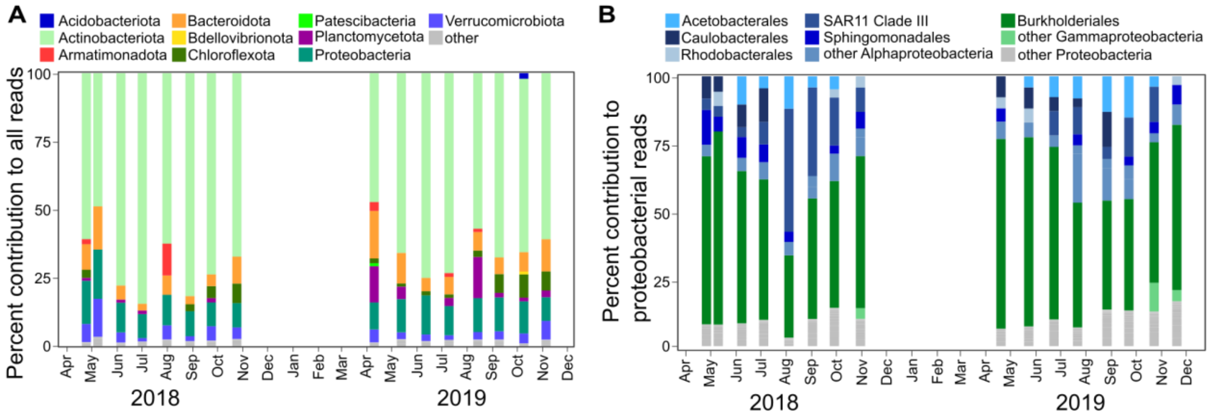

Supplementary Figure S4. Composition of bacterial communities (based on the 16S rRNA gene amplicons).

A: percent contribution of the top 10 most abundant phyla to the total number of reads in the sequencing libraries; **B:** percent contribution of proteobacterial orders to the number of reads coming from Proteobacteria.

R scripts for data analysis

Script for calculating linear mixed-effects model

```
library(nlme), packageVersion("nlme")

# importing csv file with data organized in the long format (Data S2-S7)
# activity data (rates of respiration, and of assimilation of glucose,
# pyruvate, leucine and thymidine, here referred to as 'activity_var') were
# not transformed
# environmental data for each sampling date (Data S1) were log10
transformed # and copied to each row to match the replicated activity data.
Here they are # referred to as 'envir_var'

dt <- read.csv("file_with_data.csv", header = TRUE)

# calculating linear mixed-effects models
# Date (of sampling) was considered the random variable

# model with intercept only
lme.intercept <- lme(activity_var ~ 1, random = ~1 | Date, data = dt,
method = "ML")

summary(lme.intercept)
anova(lme.intercept)

# model testing effect of light
lme.Light <- update(lme.intercept, .~. + Light)

summary(lme.Light)
anova(lme.Light)
anova(lme.intercept, lme.Light)

# models testing relationships with environmental variables one by one

lme.envir_var_i <- update(lme.Light, .~. + envir_var_i)

summary(me.envir_var_i)
anova(lme.envir_var_i)
anova(lme.Light, me.envir_var_i)

# models testing relationships with multiple environmental variables that
# were significant
lme.envir_var_i_j <- update(lme.Light, .~. + envir_var_i + envir_var_j)

summary(lme.envir_var_i_j)
anova(lme.envir_var_i_j)
anova(lme.Light, lme.envir_var_i, lme.envir_var_j, lme.envir_var_i_j)

# models testing interactions of significant environmental variables with
# light
lme.interaction.envir_var_i_j <- update(lme.Light, .~. * (envir_var_i +
envir_var_j))

summary(lme.interaction.envir_var_i_j)
anova(lme.interaction.envir_var_i_j)
anova(lme.Light, lme.envir_var_i, lme.envir_var_j, lme.envir_var_i_j,
lme.interaction.envir_var_i_j)
```

```
# calculating separate models with for the IR light and dark rates in case
of
# significant interaction with the light
dark <- dt$Light=="Dark"
IR <- dt$Light=="IR"

lme.envir_var_i.Dark <- lme(activity_var ~ envir_var_i, random = ~1 | Date,
data = dt, subset = dark, method = "ML")

summary(lme.envir_var_i.Dark)
anova(lme.envir_var_i.Dark)

lme.envir_var_i.IR <- lme(activity_var ~ envir_var_i, random = ~1 | Date,
data = dt, subset = IR, method = "ML")

summary(lme.envir_var_i.IR)
anova(lme.envir_var_i.IR)

# obtaining 95% confidence intervals of coefficients
intervals(lme.interaction.envir_var_i_j)
```

Script for 16S amplicon analysis

```
# The concatenating files and primer removal steps were done in the Linux
# environment
# Fastq files from 2 fractions per sample were concatenated into a unique
# file for forward (R1) and reverse (R2) separately for each sample
cat R*.fastq.gz > concatenated_R*.fastq.gz

# Primers were removed from forward (R1) and reverse (R2) reads using
# cutadapt v3.1

ls path_to_foleder_with_sequence_files | sed 's/ {}R._001.fastq.gz//' |
uniq | time parallel -j+0 --eta cutadapt \
-o path_to_folder_with_trimmed_sequence_files/{}R1_001.fastq.gz \
-p path_to_folder_with_trimmed_sequence_files/{}R2_001.fastq.gz \
path_to_foleder_with_sequence_files/{}R1_001.fastq.gz \
path_to_foleder_with_sequence_files/{}R2_001.fastq.gz \
-g CCTACGGGNGGCWGCAG -G GACTACHVGGGTATCTAATCC -e 0.1 -m 230

# Subsequent analysis were done using Rstudio
library(dada2), packageVersion("dada2")

# Forward (Samplename_R1.fastq) and reverse (Samplename_R2.fastq) fastq
files # in the folder (path) were assigned as a object
fnFs<- sort(list.files(path_to_folder_with_forward_trimmed_sequences,
pattern = "_R1.fastq.gz", full.names = TRUE))
fnRs<- sort(list.files(path_to_folder_with_reverse_trimmed_sequences,
pattern = "_R2.fastq.gz", full.names = TRUE))

# Sample names were extracted and a new folder ("filtered") was created
# assigning the filenames for the fastq.gz files
sample.names <- sapply(strsplit(basename(fnFs), "_"), `[`, 1)
filtFs<- file.path(path_to_new_folder for_filtered_sequences,"filtered",
paste0(sample.names, "_F_filt.fastq.gz"))
filtRs<- file.path(path_to_new_folder for_filtered_sequences,"filtered",
paste0(sample.names, "_R_filt.fastq.gz"))
names(filtFs) <- sample.names
names(filtRs) <- sample.names

# Forward and reverse reads were quality filtered and truncated at 225
```

```
# nucleotides and reads shorter than 225 nucleotides were removed
out<- filterAndTrim(fnFs, filtFs, fnRs, filtRs, truncLen = c(225, 225),
maxN=0, maxEE=c(2,2), truncQ=2, rm.phix=TRUE, compress=TRUE, multithread =
FALSE)

# Parametric error model was used to calculate the error rates
errF<- learnErrors(filtFs, multithread = TRUE)
errR<- learnErrors(filtRs, multithread = TRUE)

# Sequence variants were inferred
dadaFs<- dada(filtFs, err = errF, multithread = TRUE, pool= TRUE)
dadaRs<- dada(filtRs, err = errR, multithread = TRUE, pool= TRUE)

# Forward and reverse reads were merged together and an ASV table (seqtab)
# was created
mergers<- mergePairs(dadaFs, filtFs, dadaRs, filtRs, verbose = TRUE)
seqtab<- makeSequenceTable(mergers)

# Chimeric sequences were removed using the method consensus
seqtab.nochim <- removeBimeraDenovo(seqtab, method = "consensus",
multithread= FALSE, verbose = TRUE)

# Taxa assignment was done using SILVA r138.1 database released on August
27, 2020.
taxa_newsilva <- assignTaxonomy(seqtab.nochim,
"~/silva_nr99_v138_wSpecies_train_set.fa", multithread=FALSE,
outputBootstraps = FALSE)


# Filtering of the data was done in the phyloseq package
library(phyloseq) packageVersion("phyloseq")

# Phyloseq file construction using ASV table (seqtab.nochim), taxa
# assignmentfile (taxa_newsilva) and the sample data (samdf) that included
# sample names and dates.
ps <- phyloseq(otu_table(seqtab.nochim, taxa_are_rows=FALSE),
sample_data(samdf), tax_table(taxa_newsilva))

# Sequences belonging to Cyanobacteria phylum were excluded from the
# analysis
ps_noChloro_noCyano<- subset_taxa(ps, Phylum != "Cyanobacteria")

# Low abundant ASV, not seen more than 3 times in at least 20% of the
# samples, were excluded from the analysis
ps_noChloro_noCyano <- filter_taxa(ps_noChloro_noCyano, function(x) sum(x >
3) > (0.2*length(x)), TRUE)
```

Script for AAP bacteria analysis using PufM gene sequences

```
# AAP bacteria analysis was done following the same scripts as for 16S
# amplicons shown above, with some parameters changes as shown below.
# Cutadapt was run using the PufM primers sequence (see material and
# methods).

# Forward and reverse reads were truncate at 130 nucleotides
out<- filterAndTrim(fnFs, filtFs, fnRs, filtRs, truncLen = c(130, 130),
maxN=0, maxEE=c(2,2), truncQ=2, rm.phix=TRUE, compress=TRUE, multithread =
FALSE)

# Chimeric sequences were removed using "pooled" method
```

```
seqtab.nochim <- removeBimeraDenovo(seqtab, method = "pooled", multithread=
FALSE, verbose = TRUE)

# For the taxa assignment, a custom PufM database was used as described in
# material and methods.
taxa<-assignTaxonomy(seqtab.nochim, "~/pufM_database.fasta",
multithread=FALSE, outputBootstraps = FALSE)
```

Script for transformation of read number data for subsequent distance based redundancy analysis in Primer7 with PERMANOVA+ addon

```
library(DESeq2), packageVersion("DESeq2")

# importing ASV table with ASV in rows and samples in columns
ASV.table <- read.csv("ASV_table.csv", header = TRUE, row.names = 1)

# transforming data
ASV.VST <- varianceStabilizingTransformation(as.matrix(ASV.table),
blind = FALSE, fitType = "mean")

# changing negative values to 0s (to enable downstream analysis)
ASV.VST[ASV.VST < 0] <- 0

# exporting transformed matrix to csv
write.csv(ASV.VST, file = "ASV-VST.csv", row.names = TRUE)
```

Legends for Datasets S1 to S7

Separate csv files, columns separated with a semicolon (;) and with a comma (,) as a decimal separator.

<u>Dataset S1</u>

Physical, chemical and biological factors: Temperature (°C), total bacterial abundance (Bacteria, cells ml^{-1}), AAP bacteria abundance (AAP, cells ml^{-1}), relative AAP abundance (AAP.per, %), abundance of picocyanobacteria (picocyano, cells ml^{-1}), concentration of bacteriochlorophyll-a (Bchla, ng l^{-1}), concentration of chlorophyll-a (Chla, µg l^{-1}), concentration of dissolved inorganic carbon (DIC, mg l^{-1}), ph , concentration of dissolved organic carbon (DOC, mg l^{-1}), concentration of soluble reactive phosphorus (SRP, µg l^{-1}), concentration of nitrogen in form of ammonia (NH4-N, µg l^{-1}), concentration of nitrogen in form of nitrate (NO3-N, µg l^{-1}), and concentration of total dissolved nitrogen (mg l^{-1}).

Dataset S2

Respiration (oxygen consumption) measured in the dark and the IR light (µmol O_2 L^{-1} h^{-1})

Dataset S3

Incorporation of bicarbonate ($H^{14}CO_3^-$) measured in the dark and the IR light (µmol C L^{-1} h^{-1}).

Dataset S4

Assimilation of ^{3}H-glucose measured in the dark and the IR light (nmol C L^{-1} h^{-1}).

Dataset S5

Assimilation of ^{14}C-pyruvate measured in the dark and the IR light (nmol C L^{-1} h^{-1}).

Dataset S6

Assimilation of ^{3}H-leucine measured in the dark and the IR light (nmol C L^{-1} h^{-1}).

Dataset S7

Assimilation of ^{3}H-thymidine measured in the dark and the IR light (nmol C L^{-1} h^{-1}).
